# Alterations of specific biomarkers of metabolic pathways in vascular tree from patients with Type 2 diabetes

**DOI:** 10.1186/1475-2840-11-86

**Published:** 2012-07-24

**Authors:** Bernal-Lopez M Rosa, Llorente-Cortes Vicenta, Gomez-Carrillo Victor, Lopez-Carmona Dolores, Calleja Fernando, Gomez-Huelgas Ricardo, Badimon Lina, Tinahones Francisco J

**Affiliations:** 1 Biomedical Research Laboratory, Endocrinology Department, Hospital Virgen de la Victoria, Malaga, Spain; 2 Cardiovascular Research Center, CSIC-ICCC, Hospital de la Santa Creu i Sant Pau, Barcelona, Spain; 3 Internal Medicine Department, Hospital Carlos Haya, Malaga, Spain; 4 Cardiovascular Surgery Department, Hospital Carlos Haya, Malaga, Spain; 5 Ciber Fisiopatologia de la Obesidad y Nutricion (CB06/003) Instituto de Salud Carlos III, Madrid, Spain

**Keywords:** Atherosclerosis, Type 2 diabetes mellitus, Glycemic control, Metabolic pathways, Vascular system

## Abstract

The aims of this study were to check whether different biomarkers of inflammatory, apoptotic, immunological or lipid pathways had altered their expression in the occluded popliteal artery (OPA) compared with the internal mammary artery (IMA) and femoral vein (FV) and to examine whether glycemic control influenced the expression of these genes. The study included 20 patients with advanced atherosclerosis and type 2 diabetes mellitus, 15 of whom had peripheral arterial occlusive disease (PAOD), from whom samples of OPA and FV were collected. PAOD patients were classified based on their HbA1c as well (HbA1c ≤ 6.5) or poorly (HbA1c > 6.5) controlled patients. Controls for arteries without atherosclerosis comprised 5 IMA from patients with ischemic cardiomyopathy (ICM). mRNA, protein expression and histological studies were analyzed in IMA, OPA and FV. After analyzing 46 genes, OPA showed higher expression levels than IMA or FV for genes involved in thrombosis (F3), apoptosis (MMP2, MMP9, TIMP1 and TIM3), lipid metabolism (LRP1 and NDUFA), immune response (TLR2) and monocytes adhesion (CD83). Remarkably, MMP-9 expression was lower in OPA from well-controlled patients. In FV from diabetic patients with HbA1c ≤6.5, gene expression levels of BCL2, CDKN1A, COX2, NDUFA and SREBP2 were higher than in FV from those with HbA1c >6.5.

The atherosclerotic process in OPA from diabetic patients was associated with high expression levels of inflammatory, lipid metabolism and apoptotic biomarkers. The degree of glycemic control was associated with gene expression markers of apoptosis, lipid metabolism and antioxidants in FV. However, the effect of glycemic control on pro-atherosclerotic gene expression was very low in arteries with established atherosclerosis.

## Introduction

Cardiovascular diseases (CVD) are highly prevalent in the general population, affecting most adults over 60 years of age. Vascular endothelium has unique responses to hemodynamic forces. The flow and hemodynamic forces are not uniform in the vascular system. The endothelium of the vascular circulation is exposed to hemodynamic forces of greater magnitude than in other human tissues. Hemodynamic forces play an important role in vascular diseases, especially in the location of atheromas [1].

The pathophysiology of arterial thrombosis is different from that of venous thrombosis. In the arteries there is altered endothelium-platelet adhesion, greatly influenced by hemodynamic forces, while the major factors for venous thrombosis are the phenomena of slow or stagnant blood flow, combined with hypercoagulability situations [2].

Sustained flow with high shear stress upregulates gene and then protein expression in endothelial cells, which has a protective effect against the atherosclerotic process [3]. In the venous system, a disturbed flow leads to inflammation and venous thrombosis, and therefore the development of chronic vessel disease, such as peripheral arterial disease.

Atherosclerosis is associated with processes such as inflammation [4] lipid metabolism [5], apoptosis [6] and the immune system responses [5]. Epidemiologic studies show a consistent association between diabetes and cardiovascular disease [7]. The influence of evaluating the atherosclerosis process according to the effect of tight glycemic control has been less convincing in clinical trials [8]. Hyperglycemia could lead to vascular complications via several mechanisms. Hyperglycemia *per se* activate transcription factors that modulate the expression of a number of genes in endothelial cells, monocyte-macrophage and vascular smooth muscle cells favoring atherosclerotic process.

In this study our aims were to determine whether different biomarkers (inflammatory, oncogenic, immunological or lipid) had altered gene expression in different atherosclerotic blood vessels (artery vs. vein) and to examine whether glycemic control influenced the expression of these genes.

## Materials and methods

### Subjects

All patients were hospitalized in the Cardiovascular Surgery Department of Carlos Haya Hospital (Malaga, Spain) between February 2007 and June 2008. Those diagnosed with an advanced atherosclerotic process and type 2 diabetes mellitus were recruited to this study. Two types of vascular biopsies were collected from patients with clinical stage IV peripheral arterial occlusive disease and lower limb amputation (PAOD): 1) occluded popliteal artery (OPA) with atherosclerotic plaque and 2) femoral vein (FV). Both the OPA and the FV were obtained from the vascular package of each patient (n = 15). Control arteries with no atherosclerosis consisted of internal mammary artery (IMA) biopsies collected from 5 diabetic patients with good glycemic control and severe ischemia due to ischemic cardiomyopathy (ICM) who were undergoing coronary revascularization.

Patients were included if they were aged 18–80 years (all patients were over 60) and provided written informed consent. Patients were excluded if they had associated diseases such as alcoholism, drug addiction or HIV. Individuals who refused to participate in the study were considered losses.

We evaluated the presence of atherosclerotic risk factors using the definitions of the Spanish Society of Hypertension (blood pressure, systolic ≥140 and/or diastolic ≥90 mmHg), ADA (fasting blood glucose level ≥126 mg/dl), and the NCEP-ATP3 criteria (triglycerides ≥150 mg/dl and HDL cholesterol <40 mg/dl in men or <50 mg/dl in women). A BMI (Kg/m^2^) >30 was used for the presence of obesity and patients were considered to be smokers if they had smoked up to 6 months before hospital admission. Anthropometric and biochemical parameters included sex, age, waist circumference, systolic and diastolic blood pressure, glucose, HbA1c, total cholesterol, high-density lipoprotein (HDL) cholesterol, low-density lipoprotein (LDL) cholesterol and triglycerides. The HOMA (Homeostasis Model Assessment) index, a method used to quantify insulin resistance and beta-cell function, was also recorded. The approximating equation for insulin resistance used a fasting blood sample, and was derived by use of the insulin-glucose product, divided by a constant: (glucose x insulin)/405 where glucose is given in mg/dL and insulin is given in μU/mL.

The patients had type 2 diabetes mellitus for 12 ± 7 years. Type 2 diabetes mellitus was diagnosed according to the ADA definition (2010) by the presence of repeated fasting glucose levels ≥126 mg/dl if the patients were being treated with oral antidiabetic agents or insulin at the time of the study or if their HbA1c was >6.5%. This definition was corroborated with the patient’s medical history in order to avoid misdiagnosis due to stress hyperglycemia during hospital admission. The patients with PAOD were divided into either well-controlled or poorly-controlled groups, depending on their glucose and HbA1c (Table 1). All the diabetic patients were treated with metformin and 70% of the poorly-controlled diabetic patients were treated with insulin. The patients were admitted to the hospital 72 h before surgery. Their treatment was usually modified in order to prepare the patient for surgery. There was a drug washout period of 12 h prior to blood collection.

**Table 1:** Patient’s characteristics with CMI or PAOD and their characteristics when classified as well-controlled (HbA1c ≤ 6.5) or poorly-controlled (HbA1c > 6.5) diabetic patients

			**PAOD Subjects**	
	**CMI Subjects**	**PAOD Subjects**	**DM2 with HbA1c ≤ 6.5**	**DM2 with HbA1c > 6.5**
N (%)	5 (25.0)	15 (75.0)	6 (40.0)	9 (60.0)
Age (years)	63.2 ± 13.0	67.3 ± 14.2	73.8 ± 11.8	63.0 ± 14.6
Sex (Male/Female) (%)	5/0	12/3	5 (83.3)/2 (16.7)	7 (77.8)/2 (22.2)
Weight (Kg)	75.9 ± 10.1	68.7 ± 12.1	63.5 ± 11.3	72.1 ± 11.9
Waist circumference (cm)	94.5 ± 3.5	96.7 ± 8.7	96.5 ± 12.7	90.8 ± 19.2
BMI (kg/m^2^)	27.9 ± 3.5	25.3 ± 3.9	24.6 ± 4.4	25.7 ± 3.7
SBP/DBP (mmHg)	133/74 ± 24/14	150/76 ± 24/13	141/78 ± 21/6	155/75 ± 26/16
**Glycemia (mg/dL)**	105.2 ± 8.3	129.2 ± 48.1	96.2 ± 31.1	151.2 ± 32.6*
**HbA1c (%)**	6.2 ± 0.3	7.8 ± 1.8	6.0 ± 0.4	8.9 ± 1.3**
Creatinine (mg/dL)	1.0 ± 0.3	1.9 ± 0.9	1.2 ± 0.7	2.5 ± 1.7
Uric acid (mg/dL)	5.4 ± 1.4	4.5 ± 2.8	3.6 ± 2.0	5.3 ± 3.3
Total cholesterol (mg/dL)	135.8 ± 37.9	152.7 ± 55.6	126.5 ± 67.0	179.9 ± 61.1
LDL cholesterol (mg/dL)	81.8 ± 15.5	88.0 ± 28.12	73.4 ± 21.7	112.4 ± 52.2
HDL cholesterol (mg/dL)	32.4 ± 12.0	32.6 ± 13.8	32.3 ± 18.9	32.9 ± 9.0
Triglycerides (mg/dL)	201.0 [106.0-302.0]	122.0 [82.3-234.5]	116.5 [74.8-131.5]	210.5 [83.5-364.3]
GOT (U/l)	27.2 ± 9.8	34.23 ± 9.3	40.8 ± 46.5	28.6 ± 19.7
GPT(U/l)	53.2 ± 30.0	40.7 ±19.0	42.5 ± 20.5	39.1 ± 19.1
GGT (U/l)	30.2 ± 9.8	56.1 ± 7.1	89.3 ± 40.7	114.6 ± 132.8
Insulin (μUI/ml)	13.5 ± 9.3	11.8 ± 6.4	10.5 ± 5.8	12.6 ± 7.3
HOMA index	5.9 ± 4.6	4.1 ± 2.2	2.8 ± 1.6	5.3 ± 2.4
Hypertension (%)	21.4	73.3	66.7	77.8
Dyslipidemia (%)	57.1	85.7	83.3	87.5
Obesity (%)	16.7	33.3	33.3	33.3
Smoker (%)	33.3	13.3	11.1	22.2

Values are shown as mean ± SD (* p=0.02 vs well control; ** p=<0.0001 vs well control).

Control patients (patients with ICM) were aged 63 ± 13 years and had similar baseline characteristics as the patients with PAOD. Patient’s characteristics did not significantly differ between the groups.

The study protocol complied with the principles of the Helsinki Declaration. The study was approved by the hospital ethics committee and all the patients gave written informed consent to participate in the study.

### Isolation of human mRNA from biopsies

Samples of OPA, FV and IMA vessels were homogenized in ice using the Tripure Isolation Reagent (Roche Molecular Biochemicals, Barcelona, Spain) according to the manufacturer’s instructions using a laboratory batch mixer (T25-Ultra3-Turrax Basic; Ika^R^ Laboratory Equipment).

### Real-time PCR

A total of 46 genes were studied in these vascular biopsies. The genes were classsified depending on the metabolic pathway involved. Housekeepping genes: 18 S and GAPDH. Lipid metabolism genes: PPARg, PTGS1 (Cox1), PTGS2 (Cox2), CD36, LDLR, LRP1, NDUFA2, SCARB1, OLR1, ABCA1, TFPI, USP9Y. Apoptotic genes: CDKN1A, BCL2, BAX, Caspase 3, CD83, MMP2, MMP3, MMP9, MMP13. Signal transduction genes: CCL2 (MCP1), AKT1, VEGFA. Inflammatory genes: F3 (tF), CD34, AGER, CRP, Von Willebrand factor. Immune reponse genes: CD86, TLR2, TLR4. Cytoskeleton regulator genes: Endoglin, Actin A1, MMP10, MMP12, TIMP1, TIMP3. Translation-transcription regulator genes: SREBF1, SREBF2, Sp1, HIF1A, TP53, NKIRAS2.

cDNA was obtained from 1 μg RNA using the High Capacity cDNA Archive Kit (Applied Biosystems, San Francisco, California, EEUU). RNA purity was determined by measuring the A260/A280 ratio. RNAs with ratios between 1.7 and 2 were considered adequate for quantification of mRNA expression. cDNA synthesis was obtained from 1 μg RNA using the protocol provided with the High Capacity cDNA Reverse Transcription kit (Applied Biosystems, Foster City, CA, USA). Recombinant RNasin Ribonuclease Inhibitor (Applied) was added to prevent RNase-mediated degradation. cDNA was stored at –20°C.

Gene expression analyses were performed at mRNA level by Taqman Low-Density Array (TLDA). Pre-designed TaqMan probe and primer sets for target genes were chosen from an on-line catalogue (Applied). Once selected, the sets were factory-loaded into the 384 wells of the TLDA card which was configured into eight identical 24 gene sets in duplicate. Twenty-two genes were chosen based on literature reviews of key molecules in inflammation and immunology. Each set of genes also contained two housekeeping genes, *GAPDH* and *18srRNA.*

Five μl of single-stranded cDNA (equivalent to 100 ng of total RNA) were mixed with 45 μl of nuclease-free water and 50 μl of TaqMan Universal PCR Master Mix. After gentle mixing and centrifugation, 100 μl of mixture was transferred into a loading port on a TLDA card. The card was centrifuged twice for 1 minute at 1100 rpm to distribute the samples from the loading port into each well. It was then sealed and placed in the Micro Fluidic Card Sample Block of an Applied Biosystems 7900HT PCR system (Applied Biosystems). The thermal cycling conditions were 2 min at 50°C and 10 min at 95°C, followed by 40 cycles of 30 s at 97°C and 1 min at 59.7°C. Expression levels were measured in duplicate. Only the genes with reproducible amplification curves of both duplicates were analyzed and are presented. TLDA cards were analyzed with RQ documents and the RQ Manager Software for automated data analysis. Gene expression values (RQ) were calculated based on the ΔΔC_t_ method. Delta cycle threshold (Ct) values, defined as the point at which the fluorescence rises above the background fluorescence, were calculated with SDS 2.3 software (Applied Biosystems). A mixture of DNA from IMA was used as a calibrator and the *18srRNA* housekeeping gene was the reference for normalization.

### Western blot analysis

The proteins from IMA, FV and OPA biopsies were analyzed by western blot analysis as described previously [9]. Blots were incubated with monoclonal antibodies against human tF (ADI, Ref: 4501; dilution 1:1000; American Diagnostica Inc., Stamford, Connecticut, EEUU) and LRP-1 (RDI; PR-61067; Fitzgerald dilution 1:50; Fitzgerald Industries International, North Acton, MA, USA). Equal proteins loading in each line was verified by staining filters with Ponceau and also by incubating blots with monoclonal antibodies against β-actin (clone AC-15, Sigma). Western blot bands were quantified with a Chemi-doc (BioRad) using the Quantity One 1-D Analysis Software. Results are expressed as arbitrary units (AU) that refer to units of intensity/mm^2^.

### Histological analysis

The vascular biopsies, removed immediately after surgery, were immersed in 2-methylbutane on liquid nitrogen. Histological studies were performed on 4-mm thick sections of the vessels and atheromatous plaque thaw-mounted onto poly-L-lysine treated slides, cut in a cryostat at -20°C. Tissues were then stained with Masson’s trichrome and photographed under routine light microscopy (Leica Microsystems Ltd.).

### Statistical analysis

Results are expressed as mean ± standard deviation (SD). The baseline clinical characteristics of each group were analyzed by one-way analysis of variance (ANOVA). All probability values were two-tailed, and all confidence intervals were computed at the 95% level. Differences were considered significant if the p value was less than 0.05. Chi-square analysis was used to compare the qualitative variables.

Relationships between cell biomarkers and continuous variables were examined by Spearman correlation analysis, which measures the linear relation between two quantitative variables (−1 < r < 1). Multiple regression models were used to correct for confounding factors to assess the association between mRNA expression levels of different biomarkers, risk factors or drugs. Statistical analyses were performed with SPSS for Windows, version 11.5 (IBM Corporation INC. Somers, NY (USA)).

## Results

The baseline biological characteristics of the CMI and PAOD patients are shown in Table (Variables are mean ± SD). No significant differences were found for any of the variables studied between ICM and PAOD patients (data not shown). In contrast, there were significant differences in glycemia and HbA1c between well- and poorly-controlled PAOD diabetic patients.

### Histological characterization of vascular biopsies from atherosclerotic patients

**Figure 1:**
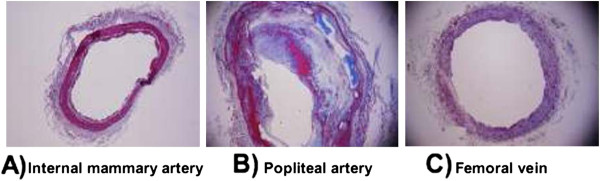
**Histological sections of arteries, internal mammary (A) and popliteal artery (where the atheroma plaque is located) (B) and femoral vein (C).** The sections were stained with Masson’s trichrome and photographed by routine light microscopy (4x).

All vessel biopsies were stained with Masson’s trichrome and photographed under light microscopy (Figure 1).

The IMA showed a normal histology with the adventitia composed of connective tissue as well as collagen and elastic fibers, the media layer composed of smooth muscle and elastic fibers, and the intima layer, the inner layer, composed of an elastic membrane lining and smooth endothelium covered by elastic tissues (Figure 1A).

The section corresponding to the OPA had soft plaques with a lipid nucleus and calcium deposits. The capsule had few collagen fibers and VSMC, although with an excess of macrophages and lymphocytes (Figure 1B).

In the FV biopsies three layers were observed: the adventitia, media, intima layer and endothelium (Figure 1C).

Both IMA and FV were composed of the same type of layers, although IMA contained elastic layers that make it more amenable to blood pressure variation.

### mRNA expression of different biomarkers in the vascular system from atherosclerotic vessels of diabetic patients

To investigate the possible alteration in gene expression of different biomarkers we analyzed mRNA levels in IMA, OPA and FV from ICM and PAOD patients.

**Figure 2:**
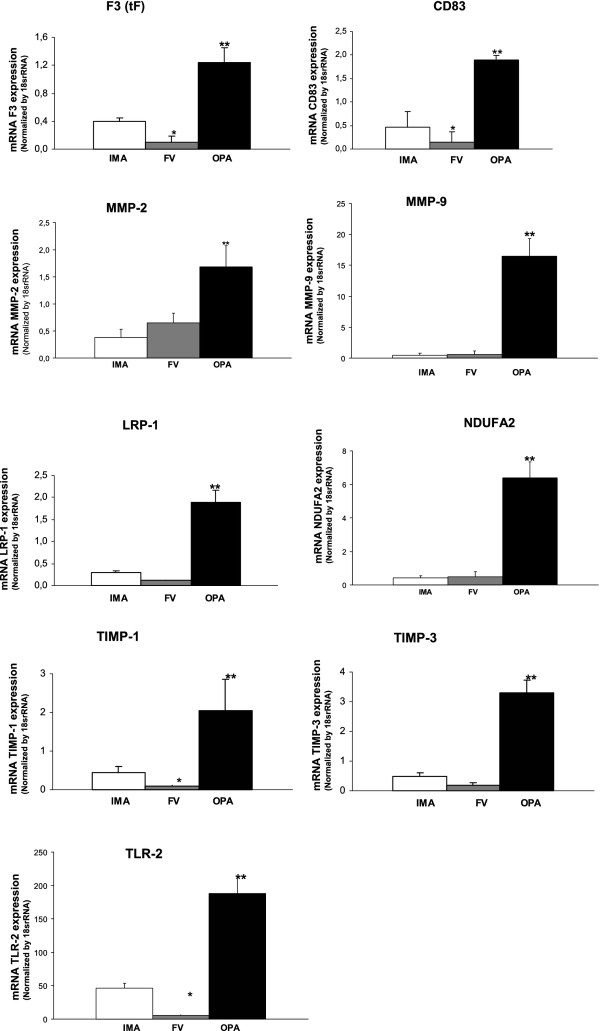
**Expression of different genes in internal mammary artery (IMA), femoral vein (FV) and occluded popliteal artery (OPA) from atherosclerotic patients with ICM or PAOD.** (* p<0.05 vs control (IMA) ** p<0.05 vs control (IMA) and femoral vein).

Expression was obtained from all the genes studied (46 genes), but only 9 genes were significantly different in all tissues. These altered genes, which showed greater levels in OPA compared to IMA or FV, were those involved in thrombosis (F3 (tF): ref. sequence: NM_001993.2, assay ID: Hs00175225_m1, amplicon length: 118), apoptosis (CD83: ref. sequence: NM_004233.3, assay ID: Hs00188486_m1, amplicon length: 104, MMP2: ref. sequence: NM_004530.2, assay ID: Hs00234422_m1, amplicon length: 83, MMP9: ref. sequence: NM_004994.2, assay ID: Hs00234579_m1, amplicon length: 54, TIMP1: ref. sequence: NM_003254.2, assay ID: Hs99999139_m1, amplicon length: 55 and TIMP3: ref. sequence: NM_000362.4, assay ID: Hs00165949_m1, amplicon length: 59), lipid metabolism (LRP-1: ref. sequence: NM_002332.2, assay ID: Hs00233856_m1, amplicon length: 64 and NDUFA: ref. sequence: NM_002488.2, assay ID: Hs00159575_m1, amplicon length: 65), and immune response (TLR2: ref. sequence: NM_003264.3, assay ID: Hs00610101_m1, amplicon length: 80). Housekeeping gene used were 18SrRNA (ref. sequence: X03205.1, assay ID: Hs99999901_s1, amplicon length: 187) and GAPDH (ref. sequence: NM_002046.3, assay ID: Hs99999905_m1, amplicon length: 122). (Figure 2).

### Protein expression of tF and LRP-1 in the vascular system from atherosclerotic vessels of diabetic patients

**Figure 3:**
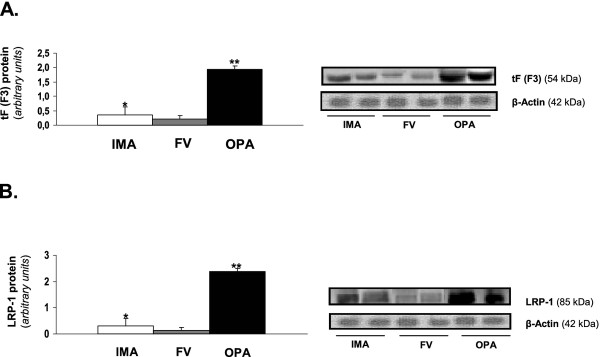
**tF (F3) and LRP-1 expression levels in human vessels from atherosclerotic patients with ICM or PAOD.A.-** Bar graphs showing the densitometric analysis of showing tF bands (results are expressed as mean ± SD. * p<0.04 vs FV or OPA, ** p<0.04 vs control (IMA) and FV) and autoradiography showing tF (54 KDa) and β-actine (42 KDa) protein levels in internal mammary artery (IMA), femoral vein (FV) and occluded popliteal artery (OPA) from two subjects from each vessel **B.-** Bar graphs showing the densitometric analysis of showing tF bands (results are expressed as mean ± SD. * p<0.05 vs FV or OPA, ** p<0.05 vs control (IMA) and FV) and autoradiography showing LRP-1 (85 KDa) and β-actine (42 KDa) protein levels in internal mammary artery (IMA), femoral vein (FV) and occluded popliteal artery (OPA) from two subjects from each vessel.

The inflammatory process and lipid metabolism are two of the key signaling pathways in the formation of atheromas in vascular wall. Therefore, and in view of the results of mRNA expression obtained of two biomarkers of these pathways (tF and LRP-1), we studied if this pattern of overexpression showed in OPA, was repeated at the protein level. We perform a Western blot analysis of both markers, confirming this effect previously observed with mRNA expression in all analyzed vessels. OPA had greater protein expression of tF than the VF and the control artery (IMA) (1.93 ± 0.12; 0.20 ± 0.12; 0.34 ± 0.23, p<0.04, respectively). The same happened when we study the levels of LRP-1. The OPA had greater protein expression of LRP-1 than the VF and the control artery (IMA) (2.38 ± 0.11, 0.14 ± 0.07, 0.30 ± 0.17, p<0.05, respectively). The results obtained are shown in Figure 3.

### Correlation analysis between anthropometric and biochemical parameters and biomarkers

Different anthropometric parameters (sex, age and blood pressure) were correlated with the genes studied. In all tissues (IMA, FV and OPA), only blood pressure showed a significant correlation with different biomarkers. In IMA biopsies, systolic blood pressure correlated negatively with MMP-9, TIMP-1, TIMP-3, PPARg and VEGFA mRNA expression (p=0.02, p=0.03, p=0.03, p=0.03 and p=0.005, respectively). FV biopsies had a positive correlation between diastolic blood pressure and HIFA1A (p=0.04). OPA biopsies showed a positive correlation between systolic blood pressure and MMP9 (p=0.03), and between diastolic blood pressure and SREBP1 (p=0.02).

Of all the biochemical parameters studied, only fasting blood glucose levels and HbA1c correlated significantly with the biomarkers studied. IMA biopsies showed no significant correlation with any of the biomarkers. However, FV biopsies showed a negative correlation between glycemia levels and BCL2, CDKN1A, Cox2 and SREBP2 (p=0.02, p=0.04, p=0.05 and p=0.03, respectively). HbA1c correlated negatively with BCL2 (p = 0.05). In OPA biopsies only the glycemia levels were influential, not the HbA1c. Fasting blood glucose levels showed a positive correlation with CD83, LRP1, NDUFA2 and TIMP1 (p=<0.0001, p=0.05 and p=0.03, respectively).

### Influence of glycemic control on biomarker expression in atherosclerotic and non-atherosclerotic vessels from type 2 diabetes mellitus patients

We classified diabetic patients into those with HbA1c ≤6.5 (good control; n = 6) and those with HbA1c >6.5 (poor control; n=9). Plasma glucose levels and HbA1c were significantly higher in the poorly-controlled patients (151.2 mg/dl ± 32.6 and 8.9% ± 1.3; p=0.02 and p=<0.0001, respectively) versus well-controlled patients (96.2 mg/dl ± 31.1 and 6.0% ± 0.4).

**Figure 4:**
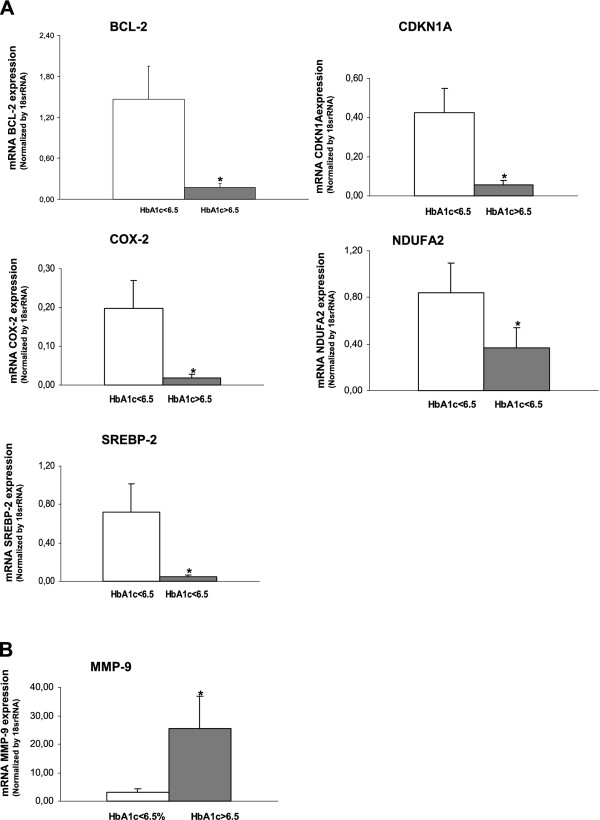
**Expression of different genes in femoral vein (A) and popliteal artery (B) from patients with PAOD, according to their HbA1c.** (* p<0.01 vs good glycemic control).

mRNA expression was obtained from FV or OPA to compare the two diabetic groups (Figure 4). In FV, the altered genes are involved in different process like apoptosis (BCL2: ref. sequence: NM_000633.2, assay ID: Hs00608023_m1, amplicon length: 81 and CDKN1A: ref. sequence: NM_078467.1, assay ID: Hs00355782_m1, amplicon length: 66), lipid metabolism (NDUFA: ref. sequence: NM_002488.2, assay ID: Hs00159575_m1, amplicon length: 65), inflammation (COX2: ref. sequence: NM_000963.1, assay ID: Hs00153133_m1, amplicon length: 75) and transcription regulation (SREBP2: ref. sequence: NM_004599.2, assay ID: Hs00190237_m1, amplicon length: 141). These genes had greater expression levels in patients with good glycemic control. However, only MMP-9 (ref. sequence: NM_004994.2, assay ID: Hs00234579_m1, amplicon length: 54) mRNA expression was increased in OPA from subjects with poor glycemic control.

## Discussion

In this study, IMA and FV had a similar gene expression pattern but very different to that seen in OPA.

Atherosclerosis is the consequence of excess lipid accumulation in vessels, which triggers an immune response, and the secretion of inflammatory cytokines that promote its development. The arteries and veins differ in that the atherosclerotic process is accentuated in large arteries where hemodynamic forces are exerted. We found significant differences between these different vessels as result of an advanced atherosclerotic process.

Lesion disruption facilitates the interaction between circulating blood and prothrombotic substances, such as tissue factor (tF) present within the atherosclerotic lesion. However, an increase of tF levels (mRNA and protein expression) was found in OPA with respect to IMA or FV, in agreement with previous studies from coronary arteries [10].

Our interest in inflammation as a component of CVD led us to study other additional mediators, such as the metalloproteinases (MMP), which form a family of zinc-dependent endopeptidases that degrade vascular extracellular matrix and basement membrane components playing a main role in tissue repair and vascular remodelling. It is widely accepted that plaque rupture plays a crucial role in the pathogenesis of vascular events and that atherosclerotic plaque destabilization is mediated by MMP. In the present study, we found higher MMP2 and MMP9 mRNA in OPA. These findings are consistent with previously published results [11]. MMP2 and MMP9 are regulated by tissue inhibitors, pro-inflammatory cytokines and other factors such as oxLDL or situations of insulin resistance, where the oxidative stress is increased [12], and they have both pro- and anti-inflammatory actions. These actions are associated with arterial stiffness and essential hypertension [13]. It was shown that MMP inhibitors and eNOS inhibit the smooth muscle cells migration *in vitro* and neointima formation *in vivo*[14]. Moreover, NO was shown to attenuate gene expression associated with insulin resistance [15].

As expected, gene expression levels of TIMP-1 and TIMP-3, regulators of the cytoskeleton, were also increased in OPA with respect to IMA and FV. TIMP-1 and TIMP-3 are endogenous inhibitors regulated by oxLDL in vascular endothelial cells [16], and are compensating mechanisms to prevent the breakdown of the atheroma plaque.

Cholesterol and LDL concentrations have indubitable value as risk markers for future cardiovascular events. Recent studies have demonstrated that increased levels of oxLDL are markedly associated with MMP-9 activation, and that statins reduce inflammatory responses. The relationship between lipid metabolism and popliteal plaque has been poorly studied. In the present study, NDUFA and LRP1 mRNA expression levels were highly increased in occluded OPA. LRP1 is upregulated by cardiovascular risk factors such as hypercholesterolemia [17] and hypertension [18]. Aditionally, this receptor contributes to the uptake of aggregated LDL [19], one of the main modifications of LDL in the arterial intima. The increase of LRP1 expression in OPA (mRNA and protein expression) suggest that LRP1 may play a crucial role in atherosclerosis progression, as previously demonstrated in other studies [20,21].

Delivery of free fatty acids excess to peripheral tissues can worsen insulin resistance and may play a role in activating inflammatory processes through activation of toll-like receptors [22]. TLRs are a family of pattern recognition receptors found in various inflammatory cells [23]. TLR2 showed increased levels in popliteal artery compared with vessels without atheroma in our study.

Different biomarkers were studied in the same environment, i.e., OPA and FV biopsies from the same diabetic patient. For this reason, it is very interesting to study what happens with the biomarkers of different metabolic pathways when the patient has good or poor glycemic control. It is likely that hyperglycemia-induced intra- and extra-cellular changes lead to alterations in signal transduction pathways, affecting gene expression and protein function and causing cell dysfunction and damage. However, in patients with type 2 diabetes mellitus, pathways involved in the diffuse vasculopathy present in non atherosclerotic arterial tissue and mRNA-alterations are already established [24].

Our results demonstrated that patients with good glycemic control had greater SREBP2 expression levels in FV. Hyperinsulinemia is related to an up-regulation of SREBPs [25], which could conflict with our results, but we previously showed that SREBP2 controls the expression of some LDL receptor genes, such as CD36 gene expression. A strong relation between SREBP2 and CD36 was found in FV but not in OPA [26]. Sampson et al. [27] showed that diabetic subjects with good glycemic control had higher CD36 expression, which could reflect a post-transcriptional efficiency of this receptor and thus there would be greater metabolism of oxLDL in these patients.

Our patients with good glycemic control showed an increased expression from genes involved in protection against apoptosis and cell turnover. Our data agree with the results recently published by Redondo et al. (2011), who demonstrated that there is a link between inflammation (COX-2) and apoptosis resistance (BCL2) in the vessels of diabetic patients [28].

Some drugs (e.g., metformin, thiazolidinediones and statins) used in the treatment of diabetes and atherosclerosis are able to up-regulate both processes. These may exert their protective effects through activation of AMPK which has potentially beneficial anti-atherosclerotic effects, such as reducing adhesion of inflammatory cells, lipid accumulation and the proliferation of inflammatory cells [29-31]. Recently, it has also been shown that adiponectin receptors ADIPOR1 and ADIPOR2, through the AMPK, may modify the risk of CVD in individuals with IGT, possibly through alterations in the mRNA expression levels [32]. Note that in this year, AMPK has been proposed as a therapeutic target for diabetic vascular disease [33].

Results from the present study show that glycemic control only exerts a significant effect on MMP-9 expession in OPA. These results are in agreement with previous studies showing that strict glycemic control does not improve cardiovascular disease progression in situations of advanced atherosclerosis [34,35].

Our study has certain limitations. The study population consists of diabetic patients with advanced atherosclerosis. Therefore, results from the present study may not be extrapolated to other population types. In addition, the sample size was small, although the significant differences found, contribute to the strength of the results.

In conclusion, when we compared occluded arteries with vessels without atheromas in diabetic and atherosclerotic patients we found significant differences in biomarkers involved in inflammation, lipid metabolism and apoptotic pathways. In addition, compensatory mechanisms could exist that prevent the rupture of the atheromatous plaque in peripheral arterial occlusive disease. On the other hand, when the atherosclerotic process is studied in terms of good or poor glycemic control into the context of diabetes, we observed that the expression of genes involved in inflammation and apoptosis protection was increased in veins from patients with good diabetic control. In contrast to veins, in arteries with advanced thrombosis, like OPA, where lumen is almost completely occluded, the glycemic control did not seem to exert any effect on gene expression profile.

## Abbreviations

ABCA1, ATP-binding cassette-family A member 1; AGER, Advanced glycosylation end product-specific receptor; AKT1, v-akt murine thymoma viral oncogene homolog 1; BAX, BCL2-associated X; BCL2, B-cell CLL/lymphoma 2; BMI, Body mass index; Casp 3, Caspase 3; CCL2 (MCP1), Chemokine (C-C motif) ligand 2; CD34, Cluster of differentiation 34; CD36, Cluster of differentiation 36; CD83, Cluster of differentiation 83; CD86, Cluster of differentiation 86; CDKN1A, Cyclin-dependent kinase inhibitor 1; CRP, C-reactive protein; CVD, Cardiovascular diseases; F3 (tF), Tissue factor; FV, Femoral vein; GAPDH, Glyceraldehyde 3 phosphate dehydrogenase; HbA1c, Glycated hemoglobin; HDL, High-density lipoprotein cholesterol; HIF1A, Hypoxia-inducible factor 1, alpha subunit; HOMA, Homeostasis model assessment index; ICM, Ischemic cardiomyopathy; IMA, Internal mammary artery; LDL, Low-density lipoprotein cholesterol; LDLR, LDL Receptor; LRP1, Low density lipoprotein receptor-related protein 1; MMP2, Metalloprotease 2; MMP3, Metalloprotease 3; MMP9, Metalloprotease 9; MMP10, Metalloprotease 10; MMP12, Metalloprotease 12; MMP13, Metalloprotease 13; NDUFA2, NADH dehydrogenase 2 alpha; NKIRAS2, NFKB inhibitor interacting Ras-like 2; OLR1, Oxidized Low Density Lipoprotein Receptor-1; OPA, Popliteal artery; PAOD, Peripheral arterial occlusive disease; PPARg, Peroxisome proliferator-activated receptor gamma; PTGS1 (Cox1), Prostaglandin endoperoxide synthase 1; PTGS2 (Cox2), Prostaglandin-endoperoxide synthase 2; SCARB1, Scavenger receptor class B member 1; Sp1, Specificity protein 1; SREBF1, Sterol regulatory element binding transcription factor 1; SREBF2, Sterol regulatory element binding transcription factor; TFPI, Tissue factor pathway inhibitor; TIMP1, Tissue inhibitor of metalloproteinase 1; TIMP3, Tissue inhibitor of metalloproteinase 3; TLR2, Toll-like receptor 2; TLR4, Toll-like receptor 4; TP53, Tumor protein p53; USP9Y, Ubiquitin specific peptidase 9, Y-linked; VEGFA, Vascular endothelial growth factor A.

## Competing interests

The authors declare that they have no competing interests.

## Authors’ contributions

MRBL and VLlC conceived the study, designed and performed the experiments and prepared the manuscript. VGC, DLC, FC and RGH collected the biochemical data from the patients. LB and FT oversaw manuscript construction and supervised the experiments. All authors read and approved the final manuscript.

## Funding

This study was undertaken with finance from FIS “Centros de Investigación En Red” (CIBER, CB06/03/0018) and REDINSCOR RD06/0003/0015 from Instituto de Salud Carlos III, Madrid, Spain.
